# Correction

**DOI:** 10.1080/15384047.2024.2299054

**Published:** 2023-12-26

**Article title**: MALAT1 promotes the proliferation and metastasis of gallbladder cancer cells by activating the ERK/MAPK pathway

**Authors**: Wu XS, Wang XA, Wu WG, Hu YP, Li ML, Ding Q, Weng H, Shu YJ, Liu TY, Jiang L, Cao Y, Bao RF, Mu JS, Tan ZJ, Tao F, Liu YB.

**Journal**: *Cancer Biology & Therapy*

**Bibliometrics**: Volume 15, Number 6, pages 806-814

**DOI**: https://doi.org/10.4161/cbt.28584

The authors recently noticed that in this article, the images in Figure 4B were inadvertently misplaced during the preparation of these figure due to their similarity. The amended version of this figure is now shown below. The conclusions of this paper are not affected. The authors sincerely apologize for this error.

**Figure 4.**
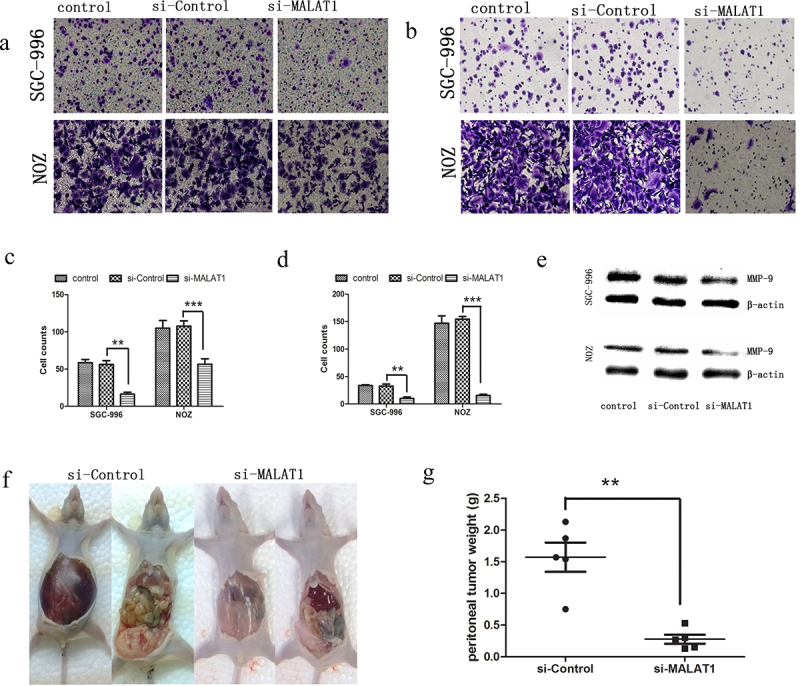
MALAT1 promoted metastasis of the GBC cells. (A) Representative photographs of crystal violet-stained SGC-996 and NOZ cells that migrated through polycarbonate membranes. (B) A statistical plot of the average number of migrated SGC-996 and NOZ cells in each group. (C) Representative photographs of crystal violet-stained SGC-996 and NOZ cells that invaded through matrigel. (D) A statistical plot of the average number of invasive SGC-996 and NOZ cells in each group. (E) Western blot analysis indicated that MMP-9 was downregulated in the SGC-996 and NOZ cells after MALAT1 knockdown. (F) A peritoneal metastasis model of human GBC was established using NOZ cells. Mice receiving the MALAT1-depleted NOZ cells exhibited little ascites at 8 wk after inoculation. (G) A statistical plot of the average tumor weights in the peritoneal metastasis model. The graph shows the mean ± SD; ***P < 0.001, **P < 0.01, *P < 0.05. The data represent one of three separate experiments. Control, blank control; si-control, cells transduced with lentivirus-mediated scr-siRNA; si-MALAT1, cells transduced with lentivirus-mediated MALAT1-siRNA.

